# 641. Clinical Predictors of Hospital-Acquired Bloodstream Infections

**DOI:** 10.1093/ofid/ofab466.838

**Published:** 2021-12-04

**Authors:** Radhika Sheth, Mehakmeet Bhatia, Vivek Kak

**Affiliations:** Henry Ford Allegiance Health, Jackson, Michigan

## Abstract

**Background:**

Hospital-acquired bloodstream infections (HABSI) are associated with increased mortality and decreased hospital quality metrics. This has led to an increased focus on blood culture stewardship. Little data exists regarding predictive factors of bacteremia in hospitalized patients. We aim to determine what clinical characteristics in patients were predictive of HABSI.

**Methods:**

This is a retrospective case-control study of 540 patients with positive blood cultures admitted to our health system between September 1, 2017, to April 1, 2020. Electronic medical records of patients with positive blood cultures were independently reviewed to determine contamination versus true bacteremia. We looked at different clinical parameters and laboratory investigations within 24 hours of drawing blood cultures. Clinical variables were age ≥ 60 years, heart rate ≥ 90/minute, systolic blood pressure ≤ 90 mmHg or use of a vasopressor, oral temperature > 38°Celsius (100.4°Fahrenheit), white blood cells (WBC) count ≥12,000/µL, lymphocytes ≤ 1000/mm3, platelets < 150,000 /µL, and creatinine >2.0 mg/dL. Stepwise logistic regression analysis was used for predictive statistical model development.

**Results:**

In a cohort of 481 patients with hospital-acquired bacteremia, 350 cases had true bacteremia and 131 cases were contaminated blood cultures. Stepwise regression analysis showed that white blood cell (WBC) count ≥ 12,000 cells/µL, lymphocyte count ≤ 1000/mm^3^, creatinine > 2.0 mg/dL, and oral temperature > 38°C (100.4°F) were associated with HABSI (R-square= 0.06, p value= 0.002).

**Conclusion:**

Our findings suggest that WBC count, lymphocyte count, creatinine, and oral temperature together can be used to develop appropriate blood culture stewardship models in the inpatient setting. This may help minimize unnecessary blood cultures.

**Disclosures:**

**All Authors**: No reported disclosures

